# Supplementary material for: Statistics or biology: the zero-inflation controversy about scRNA-seq data
Source: Genome Biol. 2022 Jan 21;23:31. doi: 10.1186/s13059-022-02601-5 (PMC8783472; doi:10.1186/s13059-022-02601-5)
Supplement: Supplementary file 2 — Additional file 2 Review history. [file 13059_2022_2601_MOESM2_ESM.docx]

Review History

**First round of review**

**Reviewer 1**

**Comments to author:**

This is a nicely written review paper that summarizes many existing literature on modeling the zero counts in single-cell RNA sequencing (scRNA-seq) studies. The paper provides a nice and comprehensive discussion on the sources of biological and non-biological zeros in scRNA-seq data. In addition, the paper also evaluates the impacts of non-biological zeros on cell clustering and differential gene expression analysis through simulations and in-depth analysis of three datasets. I enjoyed reading this paper and only have two main comments. Both comments are related to missing important references and the relevant messages conveyed in these references.

1. The paper missed an important citation on modeling zero counts in UMI data: Kim et al. (Genome Biology. 2020. 21: 196). Through analyzing multiple UMI datasets, Kim et al. provides comprehensive evidence that zero counts in the UMI data can be accounted for by either a negative binomial distribution or even simply a Poisson distribution. Kim et al. suggests that there is no need to explicitly model zero inflation for UMI count data, which is also a key message conveyed in the present review paper. In the paper, Kim et al. shows that modeling over-dispersion on top of a Poisson distribution through the negative binomial distribution is sufficient for modeling zero counts in data with a heterogeneous cell population. In addition, and perhaps more importantly, Kim et al. shows that a Poisson distribution can characterize well the sparse count data from a homogeneous cell population. Therefore, cell type heterogeneity is the main source of over-dispersion for UMI data. Consequently, how well the Poisson model fits the data can be used as a key indicator for judging the performance of cell type clustering and for guiding cell type clustering analysis. Kim et al. thus proposed a new analytic procedure HIPPO that performs iterative cell type clustering based on Poisson model fitting. Kim et al. also argues that imputing or normalizing heterogeneous data can introduce unwanted noise for UMI data analysis and are thus not recommended. These multiple messages from Kim et al. are closely related to the key messages conveyed in the current paper and thus should be discussed and incorporated into the review.

2. The paper also missed an important citation on scRNA-seq imputation for non-UMI data: Chen et al. (Genome Biology. 2018. 19:196). Chen et al. pointed out that a major drawback of scRNA-seq imputation is diminished gene expression variability across cells after imputation. The diminished expression variability across cells impacts several important downstream analysis. For example, it leads to a large number of false discoveries in the subsequent differential gene expression analysis between cell types. It can also lead to artificial cell type clusters that are incorrectly reinforced through imputation, especially when the imputation algorithm relies on cell type similarity within a cell type to carry out the imputation. Therefore, Chen et al. argued that it would be important to quantify the expression variability before and after imputation, in addition to evaluating the mean expression prediction (by, for example, comparing to the gene expression measurement in the same cell type in a separate bulk data). Because Chen et al. brought up several messages that are closely related to the paper, it would be important to discuss these potential drawbacks of scRNA-seq imputation for non-UMI data.

**Reviewer 2**

**Comments to author:**

This is a comprehensive paper, discussing the open question - zero inflation issue - in single cell studies. The authors discussed the sources of biological and nonbiological zeros in scRNA-seq data and evaluated the impacts of non-biological zeros on cell clustering and differential gene expression analysis. The advantages, disadvantages, and suitable uses of three input data types: observed counts, imputed counts, and binarized counts, are summarized very well. Finally they benchmarked the performance of the three input data types in cell clustering, dimension reduction, and differential gene expression analysis. This research would be very interesting in the relevant area.

Main concerns:

1) In Fig 6, how to simulate the DE genes? What are the parameter settings?

2) Compare more imputation methods, e.g., DCA, Enimpute etc.

3) Use more visualization methods, e.g., tSNE, EDGE, multimap.

4) Since it's very common to generate scRNA simulation data using some simulators, e.g. splatter, symsim, Sergio etc. it would be very beneficial for the future readers if the authors add an evaluation study on the impact of zeros from the popular simulators.

**Authors Response**

**Point-by-point responses to the reviewers’ comments:**

*We thank the two reviewers for their overall positive comments on our review paper. Please see below for our point-by-point responses.*

Reviewer reports:

Reviewer #1:

This is a nicely written review paper that summarizes many existing literature on modeling the zero counts in single-cell RNA sequencing (scRNA-seq) studies. The paper provides a nice and comprehensive discussion on the sources of biological and non-biological zeros in scRNA-seq data. In addition, the paper also evaluates the impacts of non-biological zeros on cell clustering and differential gene expression analysis through simulations and in-depth analysis of three datasets. I enjoyed reading this paper and only have two main comments. Both comments are related to missing important references and the relevant messages conveyed in these references.

*We thank the reviewer for the positive feedback and for pointing us to the important references we missed in our previous manuscript.*

1. The paper missed an important citation on modeling zero counts in UMI data: Kim et al. (Genome Biology. 2020. 21: 196). Through analyzing multiple UMI datasets, Kim et al. provides comprehensive evidence that zero counts in the UMI data can be accounted for by either a negative binomial distribution or even simply a Poisson distribution. Kim et al. suggests that there is no need to explicitly model zero inflation for UMI count data, which is also a key message conveyed in the present review paper. In the paper, Kim et al. shows that modeling over-dispersion on top of a Poisson distribution through the negative binomial distribution is sufficient for modeling zero counts in data with a heterogeneous cell population. In addition, and perhaps more importantly, Kim et al. shows that a Poisson distribution can characterize well the sparse count data from a homogeneous cell population. Therefore, cell type heterogeneity is the main source of over-dispersion for UMI data.

Consequently, how well the Poisson model fits the data can be used as a key indicator for judging the performance of cell type clustering and for guiding cell type clustering analysis. Kim et al. thus proposed a new analytic procedure HIPPO that performs iterative cell type clustering based on Poisson model fitting. Kim et al. also argues that imputing or normalizing heterogeneous data can introduce unwanted noise for UMI data analysis and are thus not recommended. These multiple messages from Kim et al. are closely related to the key messages conveyed in the current paper and thus should be discussed and incorporated into the review.

*We agree with the reviewer that the Kim et al. paper provides important messages that are consistent with those in our paper. We apologize for missing it in our references in the first place. Now we have cited and mentioned it in four places of our revised manuscript.*

*(1) We added the Kim et al. paper as the citation [65] to support our claim on Page 2 that “More recently, however, there were voices against the use of zero-inflated models for scRNAseq data generated by UMI protocols [64, 65].”*

*(2) We added the Kim et al. paper as the citation [65] to support our claim on Page 10 that “More recently, however, researchers have found that UMI counts are not zero-inflated when compared with the Poisson or NB distribution [6, 65, 67, 93, 94].”*

*(3) We have added a sentence to Page 10:*

*“In particular, Kim et al. provided comprehensive evidence that zero UMI counts can be accounted for by either the NB distribution or even simply the Poisson distribution [65].”*

*(4) We added the Kim et al. paper as the citation [65] to support our claim on Page 22 that*

*“In summary, the observed counts work well for UMI-based data and are robust to the introduction of non-biological zeros. As expected, the binarized counts work well under the quantile masking schemes, which largely preserve the ranks of gene expression levels. Qiu’s clustering algorithm works well for the binarized counts of UMI-based data but is not robust to the introduction of non-biological zeros. Imputation methods show concrete improvement for non-UMI-based data, but not so much for UMI-based data. This is consistent with the findings by Kim et al. that imputing UMI-based data can introduce unwanted noise and is thus not recommended [65].”*

2. The paper also missed an important citation on scRNA-seq imputation for non-UMI data: Chen et al. (Genome Biology. 2018. 19:196). Chen et al. pointed out that a major drawback of scRNA-seq imputation is diminished gene expression variability across cells after imputation. The diminished expression variability across cells impacts several important downstream analysis. For example, it leads to a large number of false discoveries in the subsequent differential gene expression analysis between cell types. It can also lead to artificial cell type clusters that are incorrectly reinforced through imputation, especially when the imputation algorithm relies on cell type similarity within a cell type to carry out the imputation. Therefore, Chen et al. argued that it would be important to quantify the expression variability before and after imputation, in addition to evaluating the mean expression prediction (by, for example, comparing to the gene expression measurement in the same cell type in a separate bulk data). Because Chen et al. brought up several messages that are closely related to the paper, it would be important to discuss these potential drawbacks of scRNA-seq imputation for non-UMI data.

*We thank the reviewer again for pointing us to the Chen et al. paper. We have now included this paper as the citation [63] in the following three places.*

*(1) On Page 2: “In early scRNA-seq data analyses, the high data sparsity provoked the use of zero-inflated models [36, 38, 46] and the development of imputation methods for reducing zeros [20, 44, 45, 47–63].”*

*(2) On Page 19: “Since the sparsity in scRNA-seq counts has posed a great hurdle for many existing tools, imputation has been proposed as a practical data pre-processing step, and many imputation methods have been developed [20, 44, 45, 47–63].”*

*(3) On Page 19: “Of course, imputation has the risk of biasing data, leading to false signals [99] or diminished biological variation [45, 63, 110]. For example, Chen et al. pointed out that a major drawback of scRNA-seq imputation is diminished gene expression variability across cells after imputation; they argued that it would be important to quantify the expression variability before and after imputation, in addition to evaluating the mean expression prediction (by, for example, comparing it to the gene expression measurement in the same cell type in a separate bulk dataset) [63].”*

Reviewer #2:

This is a comprehensive paper, discussing the open question - zero inflation issue - in single cell studies. The authors discussed the sources of biological and nonbiological zeros in scRNA-seq data and evaluated the impacts of non-biological zeros on cell clustering and differential gene expression analysis. The advantages, disadvantages, and suitable uses of three input data types: observed counts, imputed counts, and binarized counts, are summarized very well. Finally they benchmarked the performance of the three input data types in cell clustering, dimension reduction, and differential gene expression analysis. This research would be very interesting in the relevant area.

*We thank the reviewer for the summary and the positive feedback.*

Main concerns:

1) In Fig 6, how to simulate the DE genes? What are the parameter settings?

*To clarify the DE gene simulation steps and parameter settings, we have added the following sentences to Page 14:*

*“To simulate scRNA-seq data with known DE genes, we first use scDesign2 to fit probabilistic models—selected from Poisson, NB, ZIP, and ZINB models—to each cell type in each real PBMC dataset generated by Smart-seq2, Drop-seq, or 10x Genomics. Then we focus on two cell types, CD4+ T cells and Cytotoxic T cells, and define the true DE genes as the top 1500 genes that have the largest estimated mean differences (in the fitted probabilistic models) between the two cell types. We consider the remaining genes as true non-DE genes, and we set the mean parameter of each true non-DE gene to be the same for the two cell types (by averaging the gene’s estimated mean parameters in its two fitted models for the two cell types). Finally, we use scDesign2 to generate synthetic scRNA-seq data without zero inflation: for every true DE gene, we generate its synthetic counts from its two fitted models (with zero inflated components removed if existent) for the two cell types; for every true non-DE gene, we generate its synthetic counts from two altered models (with the same averaged mean, zero inflation components removed if existent, and NB’s dispersion parameters kept as they are estimated in the two fitted models).”*

2) Compare more imputation methods, e.g., DCA, Enimpute etc.

*We thank the reviewer for this suggestion, but we would like to clarify that benchmarking imputation methods is not a primary goal of our review paper, given that a comprehensive benchmark has been performed in Hou et al. (Genome BIology, 2020) [116]. Our goal is to demonstrate the importance of considering various zero generation processes (i.e., masking schemes) to achieve fair benchmarking of computational methods. Aligned with this goal, we have shown that different zero generation processes can indeed deteriorate the efficacy of different computational methods to different degrees. We have revised our text to clarify our goal and point interested readers to our GitHub code for further benchmarking.*

*Here is our newly added text to Page 20-21.*

*“To obtain the imputed counts, we use three popular imputation methods: scImpute, MAGIC, and SAVER, which demonstrate good performance in a recent study that benchmarked 18 imputation methods [118]. We note that our goal here is not to benchmark the existing > 70 imputation methods (https://www.scrna-tools.org/tools?sort=name&cats=Imputation) but to demonstrate the importance of considering various zero generation processes (i.e., masking schemes) to achieve fair benchmarking of computational methods. For readers interested in evaluating other imputation methods, we have released our code for the five masking schemes (Additional File 1) on GitHub (https://github.com/ruochenj/Five_masking_schemes).”*

3) Use more visualization methods, e.g., tSNE, EDGE, multimap.

*We have added a new figure (Fig. S4) to evaluate the performance of three visualization methods (PCA, tSNE and UMAP) on the original and imputed Smart-seq2, Drop-seq, and 10x Genomics data. We find that in terms of the average Silhouette score in the two-dimensional visualization space, UMAP has the overall best performance.*

*Here is our newly added text to Page 21.*

*“For cell dimension reduction (two-dimensional visualization), we perform PCA, t-SNE and UMAP (implemented in Seurat) on the observed and imputed counts, and then we use the average Silhouette score to evaluate how well the labeled cell types are separated in the two-dimensional space (Additional File 1: Fig. S4). We observe that UMAP has the overall best performance and thus decide to use UMAP to evaluate the impacts of imputation methods and binarization on the dimension reduction analysis (Additional File 1: Fig. S5).”*

*We have tried the dimension reduction method EDGE suggested by the reviewer but found that it was not computationally feasible for our analysis. On the Linux server (release “4.15.0-159-generic”, machine “x86_64, memory “94GB”) we used, running EDGE in Rstudio (in a single core; EDGE does not have a parallel computing option) took about 11 hours to finish for the smallest Smart-seq2 dataset with 33680 genes and 253 cells; it took too long to run EDGE on the Drop-seq and 10x Genomics datasets.*

*For MultiMAP, the method was not designed for dimension reduction but for the integration of low dimensional embeddings. In the method’s example (https://github.com/Teichlab/MultiMAP/blob/master/examples/tutorial.ipynb; code chunk [4] of the Jupyter notebook), the authors first applied PCA and then used the PCA results as the primary embeddings. Hence, we did not find MultiMAP a suitable dimension reduction method that should be included in our evaluation.*

4) Since it's very common to generate scRNA simulation data using some simulators, e.g. splatter, symsim, Sergio etc. it would be very beneficial for the future readers if the authors add an evaluation study on the impact of zeros from the popular simulators.

*We thank the reviewer for this comment. Here is our explanation of why we did not use the reviewer’s suggested simulators in our paper. In short, based on our previous study (Sun et al., Genome Biology 2021), Splatter and SymSim do not preserve genes; Sergio requires the input of a gene regulatory network and does not preserve gene-gene correlations in real data.*

*Here is our newly added text to Page 14:*

*“The reason why we use scDesign2 [102] as the simulator is that we desire synthetic cells that preserve real genes and gene-gene correlations observed in real data. The reason is that real genes are the targets of DE analysis, and synthetic cells with realistic gene-gene correlations are necessary for evaluating cell clustering and dimension reduction. As discussed in the scDesign2 paper [102], simulators such as SymSim [103] and Splatter [68] do not preserve real genes, and Sergio [104] requires an additional input of a gene regulatory network and cannot preserve the observed gene-gene correlations in real data. We note that we do not aim to benchmark simulators in this work, so we choose scDesign2, which preserves genes and gene-gene correlations and allows us to generate non-zero-inflated data, making it easy for us to introduce non-biological zeros using various masking schemes.”*

**Second round of review**

**Reviewer 1**

All my comments were well addressed.

**Reviewer 2**

The authors addressed all review comments.
